# The Control of Intestinal Inflammation: A Major Objective in the Research of Probiotic Strains as Alternatives to Antibiotic Growth Promoters in Poultry

**DOI:** 10.3390/microorganisms8020148

**Published:** 2020-01-21

**Authors:** Joan Tarradas, Núria Tous, Enric Esteve-Garcia, Joaquim Brufau

**Affiliations:** Animal Nutrition, Institute for Food and Agricultural Research and Technology IRTA, 43120 Constantí, Spain

**Keywords:** probiotic, feed additive, poultry, gut, immune system, alternatives to antibiotics

## Abstract

The reduction of antimicrobial resistance is a major challenge for the scientific community. In a few decades, infections by resistant bacteria are forecasted to be the main cause of death in the world. The withdrawal of antibiotics as growth promoters and their preventive use in animal production is essential to avoid these resistances, but this may impair productivity and health due to the increase in gut inflammation. This reduction in productivity aggravates the problem of increasing meat demand in developing countries and limits the availability of raw materials. Probiotics are promising products to address this challenge due to their beneficial effects on microbiota composition, mucosal barrier integrity, and immune system to control inflammation. Although many modes of action have been demonstrated, the scientific community is not able to describe the specific effects that a probiotic should induce on the host to maximize both productivity and animal health. First, it may be necessary to define what are the innate immune pathways acting in the gut that optimize productivity and health and to then investigate which probiotic strain is able to induce the specific effect needed. This review describes several gaps in the knowledge of host-microbiota-pathogen interaction and the related mechanisms involved in the inflammatory response not demonstrated yet in poultry.

## 1. Introduction

### 1.1. The Threats

Poultry is the main animal protein source for human nutrition, with a world production of 111.7 million tons in 2016 [1]. Antibiotics are commonly used in animal (and poultry) production, through their addition in feed and water, to treat and prevent infections and avoid the spread of diseases or as antibiotic growth promoters (AGPs). Animals account for 70% of the antibiotic consumption worldwide and a further 67% increase is expected by 2030 in response to the growing meat demand from developing countries [2,3]. Animal production is considered one of the main sources of antimicrobial resistances (AMR), thus the research for alternative strategies to the use of antibiotics in poultry are essential to fight the emergence of AMR. Currently, AMR causes the death of 700,000 people per year and it is forecasted that this will increase exponentially to 10 million by 2050, becoming the main cause of death worldwide [2]. In view of this problem, the European Union banned the use of antibiotics as AGPs in 2006 [4]. Still, poultry production will increase worldwide and the use of antibiotics will not be restricted to the same extent in all regions.

Although the reduction of antibiotics is essential to avoid AMRs, its withdrawal in poultry production will very likely result in an increased prevalence of foodborne illness-causing bacteria, such as *Salmonella* spp. or *Campylobacter* spp., on the carcass. Moreover, in-feed antibiotics have played a crucial role in the economic effectiveness of livestock production by improving the growth rate, reducing mortality, and preventing diseases [5,6,7,8]. Antibiotics directly affect the viability of pathogens and have immunomodulatory effects by interacting with the immune system and the intestinal epithelial cells (IEC), avoiding inflammation and maintaining homeostasis [9]. In addition, they can modify the microbiota population by displacing pathogenic strains and promoting intestinal colonization with beneficial bacteria [10,11,12]. Moreover, these bacteria contribute to increased energy availability and to gastrointestinal tract (GIT) epithelium restoration through the production of short-chain fatty acids (SCFA) [13,14].

An ideal alternative to antibiotics should have the same beneficial effects on the host and, therefore, similar modes of action in the GIT, microbiota, and immune system [10]. In addition, it should ensure optimal animal performance and availability of nutrients [15]. To address these challenges, a multidisciplinary approach is essential, encompassing the study of immunomodulatory agents or products with real possibilities to become alternatives to antibiotics, animal physiology (immune response and mucosal barrier), and intestinal microbiota. The research on biomarkers [16,17] to understand the mechanisms of action and the immunomodulation induced by these products on the host and their relationship with the host-microbiota-pathogen interaction is essential.

There is a considerable amount of research on the effects of probiotics on microbiota composition, integrity of the mucosal barrier, and immunomodulation to control inflammation, aiming to improve feed efficiency and performance and to reduce pathogenic infections without the use of antibiotics in poultry [10,18,19,20,21,22]. However, gaps in the knowledge make it difficult to study and select the most relevant modes of action of probiotics in the GIT. This review focused on identifying the lack of knowledge about the different innate immune pathways that act in the gut, which are indispensable to understand the modes of action of probiotics in the host to improve the gut health, performance, and welfare of chickens without the need of antibiotics.

### 1.2. The Role of Inflammation

The intestine is a structurally complex organ that performs the key roles of nutrient absorption and tolerance of innocuous/beneficial microorganisms, while retaining the ability to respond appropriately to undesirable microbes or microbial products, preventing their translocation to sterile body compartments [23]. Recently, gut health (described as gastrointestinal functionality) has been characterized as the balance between diet, effective digestion and absorption, normal and stable microbiota, effective immune status, gut mucosa, and neuroendocrine and motor function of the gut [16]. When one of these domains is altered, an intestinal dysfunction may appear, inducing the loss of homeostasis. This dysfunction in the gut health is associated with shifts in the composition of intestinal microbiome (dysbiosis), leakage of the mucosal barrier, and, finally, inflammation [17].

In a state of homeostasis, the intestinal immune system acts as an active guardian by preventing or modulating the response to a known or innocuous antigen [24]. The tolerance response is mediated by anti-inflammatory cytokines (e.g., transforming growth factor (TGF)-β and interleukin (IL)-10) and causes the inactivation of the nuclear factor-kappa B (NF-κB) pathway, responsible for inflammation induction [25]. Tolerance status promotes the expression of secretory IgA (sIgA), capable of confining microorganisms in the lumen and also mucins, reinforcing the layer of mucus that covers the epithelial barrier [26]. 

On the other hand, inflammation is the most prevalent manifestation of host innate defense in reaction to alterations in tissue homeostasis [27] (infections, host damage, and danger signaling molecules) and it is essential for maintaining homeostasis and restoring functionality [28]. However, the intestine is extremely sensitive to damage mediated by its own immune responses and, normally, inflammation ends up being the major cause of intestinal pathogenesis [29,30]. Moreover, pathogens or other external stressors can induce the activity of ineffective immune responses that are unable to control infection, allowing host tissue damage and causing inflammation [31].

The inflammatory response is mainly initiated after the detection of microbe-associated molecular patterns (MAMP) by means of pattern recognition receptors (PRR) of IEC and/or circulating immune cells. Next, the mechanisms of innate and acquired immune response, initiated and orchestrated by the activation of NF-κB pathway [25] and mediated by pro-inflammatory cytokines (e.g. IL-6, IL-8, IFN-γ), are activated, increasing the infiltration of heterophils and recruiting natural killer (NK) cells, macrophages, and TCRγδ T cells [30]. Although this response can be effective against pathogens, a prolonged inflammatory process may have undesirable consequences, including potentially exacerbating tissue damage and diverting nutrients away from productive purposes [24]. Fever and inflammation consume considerable resources, decreasing carbohydrate reserves and catabolizing proteins, and, therefore, negatively influencing animal performance [32,33]. In addition, the release of pro-inflammatory cytokines causes the syndrome of “malaise” in birds [34], causing pyrexia, anorexia, weight loss, and apathy [31]. Because of these negative effects, a strong inflammation response is especially detrimental for productivity in broilers due to their short growing cycle (35–42 days).

### 1.3. The Role of Probiotics on the Control of Inflammation

**Figure 1:**
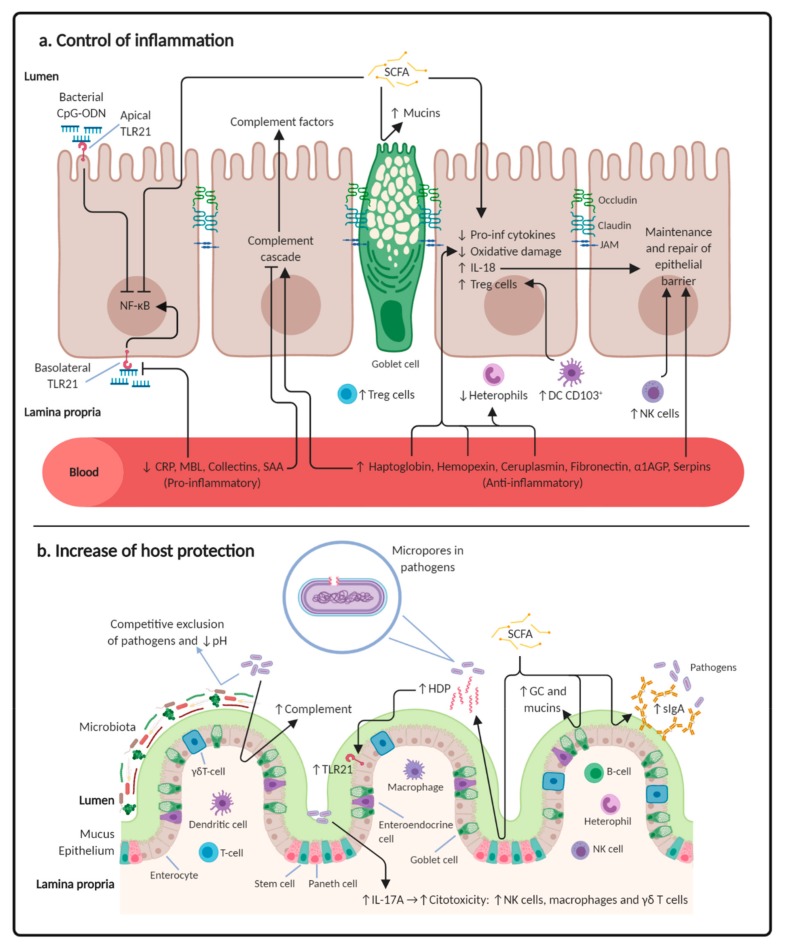
Putative mechanisms and immune pathways modulated by probiotics in the gastrointestinal tract of chickens: (**a**) by inducing a robust homeostatic status through promoting tolerance and preventing inflammation; (**b**) by increasing the luminal defence capacity of the host against pathogens. CpG oligodeoxyunucleotide sequences (CpG-ODN); nuclear factor-kappa B (NF-κB); toll-like receptor (TLR); short-chain fatty acid (SCFA); interleukin (IL); dendritic cell (DC); natural killer (NK); C-reactive protein (CRP); mannose-binding lectin (MBL); serum amyloid A (SAA); α1-acid glycoprotein (α1AGP); secretory immunoglobulin A (sIgA); host defence peptide (HDP); and goblet cell (GC). Figure created with BioRender.

Beneficial bacteria play a key role in limiting the direct contact of pathogenic bacteria with the epithelium by competitive exclusion for nutrients and for lumen surface and by generating a hostile environment (e.g., reducing intestinal pH through lactic acid production) [30]. Probiotics also have the ability to modulate inflammatory pathway activation by interacting with the intestinal epithelial and immune cells. These cells detect microbial fragments through PRRs. Toll-like receptors (TLR) are a major class of PRRs and recognize both antigens derived from the microbiota and antigens from invading pathogens. While they maintain immune tolerance to the communities of resident commensal bacteria, they mount robust immune responses against pathogens (Figure 1) [35,36]. 

Moreover, commensal and probiotic bacteria regulate homeostasis, inducing the expression of sIgA. Secretory IgA promotes the clearance of antigens and pathogenic bacteria from the lumen by blocking their access to epithelial receptors, entrapping them in mucus, and facilitating their removal by peristaltic and mucociliary activities [37]. Beneficial bacteria can also increase mucin synthesis and the number of goblet cells (GC) in the intestinal epithelium [38]. Mucins (produced by GC) are large glycoproteins that cover the epithelial surfaces of the intestine and form a mucus layer to protect IEC from gut health challenges [39]. Beneficial bacteria could also aid in the regeneration of the epithelium via PRRs during pathogenesis, the stimulation of growth factor synthesis, and the recruitment of regulatory T cells (Treg) [40] that promote tolerance in the gut.

On the other hand, dietary fiber fermentation by some commensal and probiotic bacteria generates SCFAs, which are absorbed by gut cells and used as energy source for their metabolism. Short-chain fatty acids, mainly acetate, propionate, and butyrate, are believed to play a beneficial role in gut health [41]. It has been demonstrated that butyrate inhibits NO production and reduces the expression of cytokines, such as IL-1β, IL-6, IFN-γ, and IL-10, in lipopolysaccharides (LPS)-stimulated cells [42]. It also influences the expansion and function of hematopoietic and non-hematopoietic cells [14] and inhibits the transcription of factor NF-κB, reducing the production of inflammatory cytokines [43]. In addition, SCFAs promote the production of mucus by GC, induce sIgA, and activate inflammasomes resulting from the expression of IL-18 [14,43,44]. It has been observed that IL-18, also induced by the presence of SCFAs, has a reparative and maintenance effect on the epithelial barrier [45]. Moreover, SCFAs are capable of recruiting Treg cells (increase in tolerance) and inducing the expression of host defense peptides (HDP) via histone deacetylase inhibition, enhancing the antibacterial activity and bacterial clearance of macrophages [46,47].

## 2. Effects of Probiotics on the Immune System

### 2.1. Effects of Probiotics on Acute-Phase Response

Inflammation is accompanied by systemic and metabolic changes, collectively referred to as the acute-phase response (APR). Acute-phase proteins (APP) are proteins whose plasma concentrations change by 25% or more following an inflammatory stimulus [48]. These proteins have the ability to participate in host adaptation or defense (e.g., C-reactive protein (CRP), mannose-binding lectin (MBL), fibrinogen, or serum amyloid A (SAA)) and to act as transport proteins with antioxidant activity (e.g., haptoglobin, hemopexin, or ceruloplasmin) (Figure 1). Some of them increase the inflammation reaction in response to a variety of bacteria and to intracellular antigens, activating complement and phagocytic cells (CRP, MBL, collectins, SAA). Meanwhile, other proteins have anti-inflammatory effects that protect the integrity of tissues (serpins), neutralize the toxicity of hydrophobic molecules like LPS (α1-acid glycoprotein, fibronectin), or prevent oxidative stress (haptoglobin, hemopexin, ceruloplasmin) [48] (Table 1). Even though the APR clearly has a relevant role in inducing inflammation, there are very few studies assessing the effect of probiotics on APP expression.

Haptoglobin is crucial for the elimination of free hemoglobin and the neutralization of oxidative damage and it plays an important inhibitory role on inflammation. Haptoglobin is rapidly increased in the blood by infectious agents or many physiologic changes. The effect of *Bacillus subtilis* supplementation on the serum haptoglobin concentration of birds challenged with *Salmonella typhimurium* has been studied. Supplementation with *Bacillus subtilis* (strains RX7 + B2A) resulted in a lower serum haptoglobin concentration compared with the challenged control group without probiotics, probably due to suppression of the tissue lesions caused by *Salmonella typhimurium* [49]. However, the haptoglobin concentration was not influenced by the strain B2A administered in non-challenged animals [50]. This indicates an indirect effect of probiotics on haptoglobin concentration due to a successful competitive exclusion of *Salmonella typhimurium* using *Bacillus subtilis* [49].

Another acute-phase marker that has been assessed is ceruloplasmin. This APP is a cooper-carrying protein that oxidizes toxic iron to its non-toxic ferric form. It can act as an anti-inflammatory agent, reducing the number of neutrophils, and as a scavenger of peroxide [51]. It has been observed that ceruloplasmin is not affected by *Lactobacillus salivarius* strain AWH or *Bifidobacterium animalis* strain 30 in non-challenged animals, but these probiotics reduced its concentration by up to 32% when the animals were challenged with *Salmonella*. In contrast, in animals fed avilamycin, both *Salmonella* non-infected and infected chickens showed increased levels of ceruloplasmin by up to 35% [52]. This study demonstrated that avilamycin growth promoter lowered immune response and stimulated the ceruloplasmin in the blood of chickens, but *Lactobacillus* and *Bifidobacterium* increased the immune defense markers without elevating ceruloplasmin. This correlation shows a distinct effect on immunomodulation between probiotics and AGP, which may affect different mechanisms in the immune system and in nutrition-related metabolism [52]. 

Blood C-reactive protein has also been shown to increase in response to inflammation, infection, or tissue damage [53]. C-reactive protein has the ability to bind with phosphocholine molecules expressed on the surface of dead or dying cells and thus activates the complement system [54]. A higher percentage of CRP-positive birds has been observed under heat stress (53.3%) compared with animals under thermoneutral conditions (20%). Supplementation of the heat-stressed birds with a *Lactobacillus*-based probiotic protected them against heat stress, as suggested by the decrease in the percentage of CRP-positive birds (40%) [55].

Mannose-binding lectin (MBL) also plays an important role in the innate immune response by interacting with mannose-rich residues, which are present on the surface of pathogens [56]. Through this binding, MBL activates complement, via the lectin-dependent pathway, and directly enhances opsonophagocytosis against invading pathogens [57]. A recent in vivo study showed a direct correlation between the serum concentration of MBL and the colony-forming unit counts of *Salmonella enterica* in cloacal swabs [58]. The control or induction of high MBL levels would be interesting to study during the first days of life, when the animal has not yet completed its intestinal microbiota colonization and the immune system is still immature. However, research studies assessing the modulation capacity of probiotics on collagenous lectins expression is missing. 

Moreover, it has been demonstrated that MBL downregulates non-methylated CpG oligodeoxyunucleotide sequences (CpG-ODN)-triggered TLR9 activation in human cells. MBL protein suppresses activation of NF-κB signaling and subsequent production of proinflammatory cytokines from human monocytes cells induced by CpG-ODN motifs [59]. However, CpG-ODN motifs are potent immunomodulators of bacteria and the frequency of these sequences in their genome is directly proportional to the capacity to modulate inflammation. Some strains of *Enterococcus faecalis*, *Lactobacillus casei*, *Lactobacillus plantarum*, and *Lactobacillus rhamnosus* that have been marketed as probiotics have high counts of GTCGTT motifs [60]. This suggests that some strains with probiotic effects could act as inflammation inductors, but more research is needed to understand the effects of bacterial CpG motifs on cell surface receptors of immune cells and IEC (more details in Section 2.3).

### 2.2. Effects of Probiotics on Complement

The complement system refers to soluble proteins, cell surface complement receptors, and regulatory proteins able to enhance phagocytosis and induce inflammatory response and cytolysis. Complement molecules act as a cascade and link innate and adaptive immunity. There are at least three routes of activation: the classical pathway (activated by antigen-antibody complexes), the lectin pathway (activated by MBL/ficolins binding mannose residues on microbial surfaces), and the alternative pathway (spontaneous hydrolysis of C3 into C3(H_2_O)). The cleavage of major complement factors generates the key component C3, initiating the biological functions of the complement system [48]. 

Some studies have demonstrated the presence of complement proteins in the gastrointestinal tract of mammals and birds (reviewed in [61]). Proteins like C3b, membrane attack complex (MAC), C3, and C4 were detected in the mucosa of humans with intestinal inflammatory diseases, suggesting that many complement proteins are present in the uninflamed mucosa and the complement system is activated during inflammatory conditions (Figure 1, Table 1). Even so, there are very few studies analyzing the effects of probiotics on the local or systemic complement system in mammalian and/or birds. 

A *Lactobacillus jensenii* TL2937 strain has been shown to be able to decrease the expression of complement factors C1R, C1S, C3, and CFB in porcine intestinal epithelial (PIE) cells, supporting the immunoregulatory and anti-inflammatory effect of this strain [62]. Another study addressed the response of the intestinal epithelial cell line HT29 (human colon adenocarcinoma cell line) to the strain *Bifidobacterium breve* IPLA20004. In the array of 84 genes involved in inflammation tested, the expression of 12 genes was modified by bifidobacteria, with a marked upregulation of the complement component C3 [63]. In poultry, dietary supplementation of laying hens with *Clostridium butyricum* in the late phase of production demonstrated that the addition of this probiotic resulted in an increase of serum complement component C3 [64]. These results indicate a strain-specific effect on C3 expression, differently modulating the inflammation induced by the presence of complement proteins. 

Currently, the presence of complement proteins in the intestinal epithelium/mucosa is poorly understood. Intestinal epithelial cells will synthesize some complement molecules and, during inflammation, the remaining proteins will be provided by infiltrating cells and/or blood, activating complement pathways [61]. Undoubtedly, there is a lack of knowledge about the implication of complement on intestinal inflammation, especially in healthy animals. Complement is a strong inflammation inductor and the dietary administration of probiotics may modulate this response. However, more research is needed to understand the role of complement on the homeostasis disruption in the GIT of mammals and birds and the capability of probiotics to ameliorate this effect.

### 2.3. Effects of Probiotics on TLR Repertoire

Intestinal epithelial cells provide a physical and chemical barrier between the intestinal lumen and the lamina propria. These cells detect MAMPs via PRRs and are responsible for maintaining oral tolerance to the communities of resident commensal bacteria while also being capable of mounting immune responses against pathogens [65]. Toll-like receptors are a major class of PRRs expressed on IECs and immune cells, which are involved in the induction of both tolerance and inflammation [36]. Toll-like receptors recognize a wide range of microbial fragments and therefore detect both antigens derived from the microbiota as well as invading pathogens. In avian species, ten TLRs have been described [35,66]. Some avian TLRs conservatively recognize the same ligands as mammalian TLRs (TLR4-bacterial LPS; TLR5-flagellin; TLR3-sRNA; and TLR7 ssRNA) [67]. Others were reported to form distinct paralogues with related ligand specificity (heterodimer-forming TLR1A/TLR1B together with TLR2A/TLR2B - di/triacylated lipopeptides) [68,69] or achieve recognition of similar ligands as their mammalian analogues through convergence (avian TLR21 similarly recognizes CpG DNA as mammalian TLR9) [70,71]. Finally, TLR15, which is unique to birds, evolved to gain a novel function in recognition of extracellular proteases [72].

Avian TLR4 is the main receptor of LPS, which is a major component of the outer membrane of Gram-negative bacteria [73]. After binding to LPS, avian TLR4 triggers a cascade of inflammation responses via the myeloid differentiation factor 88 (MyD88)-dependent signaling pathway, which results in NF-κB activation [67]. The increase of TLR4 expression is associated with an induction of high levels of nitric oxide production and the expression of pro-inflammatory cytokines in the intestine, although chickens are relatively resistant to LPS [48]. On the other hand, IECs showed a low expression of TLR2 and TLR4 in homeostatic conditions, causing a low sensitivity to foreign stimuli [74] and indicating the relevant role of these receptors in the induction of inflammation (Table 1).

In broiler chickens, probiotic-supplemented (*Lactobacillus fermentum* and *Saccharomyces cerevisiae*) diets increased the mRNA expression levels of TLR2 and TLR4 in the foregut, but they did not change TLR7 expression when compared with the control group [75]. Moreover, an augmented expression of TLR4 in *Lactobacillus plantarum*-supplemented groups in the ileum of laying hens has been observed [76]. However, a blend of yeast-derived carbohydrates and probiotics (*Lactobacillus acidophilus*, *Lactobacillus casei*, *Streptococcus faecium*, and *Bacillus subtilis*) downregulated the expression of TLR4 in the cecal tonsils [77]. Another study demonstrated that *Enterococcus faecium* AL41 was not able to modulate the expression of TLR4, but a boost was observed when the animals fed that probiotic were challenged with *Campylobacter jejuni* [78]. However, in an in vitro study, TLR4 mRNA expression of HT29 intestinal epithelial cells was not influenced by the presence of *Lactobacillus plantarum* [79]. Although some authors consider the results regarding TLR4 expression after probiotic administration to be contradictory [76], an explanation could be the capacity of each specific strain to up- or down-regulate the TLR4 expression. The beneficial effects could be induced through different pathways and may be due to both pro- and anti-inflammatory processes. Thus, further investigation needs to be performed to understand TLR4 regulation and the immune pathways influenced by its activation.

Another interesting TLR to take into account is TLR21. In mammals, the increased expression of TLR9 (homologous in function to TLR21 in birds) in the basolateral cells surface (lamina propria) is associated with the induction of inflammation, but the expression of TLR9 in the apical cell surface (lumen) has been described as contributing to homeostasis [25,80,81] (Figure 1, Table 1). Apical TLR9 activation prevents the degradation of IκB-α proteins and, therefore, suppresses the production of pro-inflammatory cytokines induced by the NF-κB pathway. However, the basolateral TLR9 stimulation leads to the activation of this pathway, inducing the release of IL-8 (pro-inflammatory cytokine) [60]. It has also been shown that the activation of apical TLR9 induces the secretion of galectin-9 protein, which is directly related to the differentiation of Treg cells and tolerogenic dendritic cells (DC) [82]. TLR9 recognizes bacterial DNA CpG-ODN sequences; the presence of this DNA in the lumen does not pose a real threat for the organism and a powerful immune response is not induced to eliminate it. On the other hand, the detection of bacterial DNA in the lamina propria indicates a potentially dangerous infection (bacterial translocation of epithelium) that must be combated through the activation of inflammatory pathway NF-κB.

Recently, the in vitro stimulation of mononuclear cells from chicken cecal tonsils with CpG-ODN sequences has been shown to induce an increase of TLR21 and pro-inflammatory cytokines expression probably due to the activation of the NF-κB pathway [71,83], but probiotic strains that contain suppressor motifs (TTAGG or TCAAGCTGA) can revert this situation by maintaining the concentration of Treg cells [84]. In the same way, another study demonstrated a synergy between CpG-ODN and dsRNA in promoting inflammation (Th1-biased immune response) through co-stimulation of TLR21 in chicken monocytes [85], showing a similar function of TLR9 and TLR21. Even so, these studies have not taken into account the possible in vivo anti-inflammatory activity of TLR21 when this TLR is expressed apically by the IECs. The anti-inflammatory activity of TLR21 in the apical surface has not yet been demonstrated in birds and could be a key receptor to maintain the homeostasis in the intestine.

Furthermore, it has been reported that different chicken lines showed different patterns of TLR gene expression [86,87,88]. The different basal expression patterns of TLRs’ mRNAs could indicate a host effect and, probably, a different modulation of immune system induced by the same probiotic. 

### 2.4. Effects of Probiotics on Cells of the Innate Immune System

Most immune cells in the intestine are distributed throughout the lamina propria and in the lymphoid aggregates. Different immune cell populations, such us heterophils, natural killer (NK) cells, macrophages, dendritic cells (DC), and T and B cells, take up the lamina propria whilst intraepithelial lymphocytes (IEL) (mostly γδ-T cells) are located among the intestinal epithelial cells (IEC) (reviewed in [89]) (Figure 1). 

In contrast to mammals, tissues, such as spleen, blood, or lung, of chickens contain a very low frequency of NK cells (ranging from 0.5% to 1%), being mainly confined in the intestinal epithelium [90]. These cells not only have the ability to kill infected or malignant cells, but also mediate cytotoxicity on several types of activated immune cells, playing a key role in the control of immune responses and maintenance of homeostasis [91]. A therapeutic effect of *Lactobacillus plantarum* has been detected in vitro on NK cells. This probiotic strain efficiently increased protein levels of the natural cytotoxicity receptor (NCR) family and the expression levels of IL-22 mRNA and protein in NK cells (NCM460 cells), attenuating the damage induced by enterotoxigenic *Escherichia coli* (ETEC) and protecting the integrity of the epithelial cell barrier [92]. However, the new findings on these important physiological roles of NK cells in both promoting inflammation and exerting immunoregulation and maintenance of immune homeostasis have not been studied in poultry yet. 

Recently, it has been demonstrated that not only NK cells have spontaneous cytotoxicity without prior sensitization by antigen in the intestine. Chicken γδ T cells represent the major cytotoxic lymphocyte subset that can lyse target cells in a major histocompatibility complex (MHC)-unrestricted manner in the intestine [93]. Little is known about the role of these innate killer cell populations within IEL triggered by different inflammatory stimuli and their influence on downstream antimicrobial immunity and mucosal inflammation. A mechanism that can explain the cytotoxicity of these cells is the expression and release of IL-17A by NKs, γδ T cells, and macrophages (Figure 1, Table 1). It has been demonstrated that these cell populations are able to express this cytokine after microbial invasion, being an essential cytokine in host defense at mucosal barriers [94]. IL-17A induces a fast recruitment and activation of neutrophil granulocytes or macrophages as well as granulopoiesis by the expression of pro-inflammatory cytokines in various cell types [95]. There are few studies assessing the induction or suppression of IL-17A expression after probiotic administration. An anti-inflammatory effect of *Lactobacillus fermentum* IM12 has been observed, avoiding the increase of IL-17A in mice after challenge with LPS [96], but, contrarily, a robust increase of this cytokine was detected in human peripheral blood mononuclear cells (PBMC) after their incubation with inactivated probiotic *Bacillus coagulans* GBI-30 [97]. Taking into account that the control of the expression of IL-17A is strain specific, this cytokine could act as a biomarker of inflammation control, but more research is needed to confirm this hypothesis. 

Another relevant mechanism in the control of inflammation is tolerance. Local or systemic inflammation also occurs when tolerance to luminal antigens, usually not harmful, is lost (not recognized) [65]. After the antigenic recognition, IECs and immune cells express pro- or anti- inflammatory cytokines, which contribute to GIT tolerance promoting the differentiation of tolerogenic DCs CD103^+^ able to drive the development of Foxp3^+^ T regulatory (reg) cells [98,99]. Dendritic cells CD103^+^ induce the secretion of thymic stromal lymphopoietin (TSLP), TGF-β, and vitamin A, promoting the maturation and recruitment of Treg antigen-specific cells through the expression of chemotactic molecules in the intestine (integrin α4β7 and CCR9) [100]. The expression of these chemotactic molecules could be driven by γδ T cells [101]. Regulatory T cells control the immune response through cell-cell contact or through the secretion of anti-inflammatory cytokines (IL-10 and TGF-β) [102]. The induction of this cell type is essential for mucosal tolerance since it reduces hypersensitivity to food [80] and could promote the excretion of sIgA, limiting microbial interaction with the epithelium [103]. Recently, an increased proportion of Foxp3+ Treg cells has been observed in the intestine of healthy mice after the administration of *Lactobacillus reuteri* DSM 17938 [104] and this could probably be related to an enhanced tolerance to inflammatory stimuli. Whether this mechanism occurs in poultry and the relationship between the administration of probiotics and the prevention or reduction of inflammation after an external challenge remains unknown.

### 2.5. Effects of Probiotics on Antimicrobial Peptides (AMPs) Production

The induction of a homeostatic (tolerogenic) status in the GIT could be a strategy to optimize intestinal health (and efficacy and well-being) of birds. Even so, pathogens, such as *Salmonella*, use this same mechanism to persist in the intestine and promote its spread [47]. Therefore, it is essential to consider the use or the induction of the expression of substances with antimicrobial effects to increase the protection capacity in poultry.

Antimicrobial peptides are a potent alternative to antibiotics due to their antimicrobial activity (Table 1). The activity of these small, positively-charged peptides is mediated through the disruption of bacterial membranes and they are especially interesting because the development of resistance is more difficult compared with traditional antibiotics that target enzymatic processes [105,106]. These peptides are part of the non-specific defense system and they can be produced by plants, animals, insects, and microorganisms, although they can also be synthesized chemically [107]. Probiotics can produce their own AMP (bacteriocins) [108,109]. The use of bacteriocin producers as probiotics may be cost-effective and could target specific pathogens without affecting beneficial bacteria. However, several conditions must be met, including the successful colonization of the digestive tract by the bacteriocin-producing bacteria and the actual production of bacteriocins in this environment [110].

In mammals, most of the endogenous AMP (host defense peptides—HDP) modulate innate immunity without harmful inflammatory responses [111]. It is now known that the chicken genome encodes a total of 14 avian β-defensins (AvBD1-14), 4 cathelicidins, NK-lysin, and LEAP-2 in a wide range of tissues, including digestive, respiratory, and immune systems [112,113,114]. In poultry, the expression pattern of these peptides has recently been determined for the GIT, with AvBD-14 being the constitutively most expressed in cecal tonsils [113]. When animals are challenged with *Salmonella enterica*, an increase in AvBD-3 and AvBD-4 is observed. AvBD-3 gene expression is significantly upregulated in the cecum and downregulated in the ileum, possibly reflecting the site of colonization in the cecum and the host’s response to that. The expression of AvBD-4 is upregulated in the duodenum upon *Salmonella enterica* challenge and may be important in encountering initial bacterial intestinal colonization [115]. On the other hand, chicken AvBD-8 protein is strongly expressed in white leghorn chicken intestine and in macrophages after LPS challenge and interacts with other AvBDs and antimicrobial proteins, such as leukocyte cell-derived chemotaxin-2 (LECT2) and cathelicidin-2 (CATHL-2) [116]. Moreover, it has been described that AMPs are also capable of modulating the activation of TLRs. An in vitro study demonstrated that chicken CATHL-2 activates the endosomal TLR21 from macrophages, increasing their inflammatory capacity [117]. However, its effect on the apical TLR21 activation (anti-inflammatory effect) has not been demonstrated in birds (Figure 1).

Recently, it has been observed that orally administrated probiotics also can induce the expression of HDP in pigs. A study suggested that *Lactobacillus reuteri* I5007 could modulate intestinal HDP expression and improve the gut health of neonatal piglets, probably through the increase in colonic butyric acid concentration and the up-regulation of the downstream molecules of butyric acid, PPAR-γ, and GPR41, but not through modifying gut microbiota structure [118]. However, the induction of HDP by the presence of probiotics has not yet been demonstrated in poultry.

## 3. Conclusions

To date, research on probiotics has focused on deciphering the immunomodulatory effects that each bacterial strain induces on the host, with the aim of improving productivity, feed efficiency, health, and welfare. Despite knowing and understanding the modes of action of some probiotic strains, the scientific community has not been able to describe the specific effects that a probiotic should induce on the host to maximize productivity and promote optimal health and welfare.

A more adequate approach may be to first understand the different innate immune pathways that act in the gut, identify those that optimize gut health and performance, and then to investigate which probiotic strains are able to induce the specific effects needed. Until recently, this approach had not been possible due to technological limitations. However, with the evolving “-omics” methodologies, a large body of data about immune and metabolic pathways of host and microbiota will become available in the near future, which should allow for the deciphering of the key mechanisms involved in inflammation. The selection of feed additives should then be conducted specifically for the mode of action desired, knowing exactly the effects to be induced in the host.

Although this review described several mechanisms that may be involved in this response, many of them have not been demonstrated in poultry yet. Considering the concepts reflected in this document, we hypothesized that an induction of a robust homeostatic status in the chicken GIT that promotes tolerance and prevents inflammation (e.g., Treg cell chemotaxis, activation of apical TLR21) and avoids the diversion of nutrients away from productive purposes may be a good strategy to improve efficiency. In parallel, an increase of the luminal defense capacity against pathogens (expression of HDP) and epithelial barrier protection (mucins, sIgA, and SCFA) should be induced to decrease the dependence on the use of antibiotics for efficient poultry production without compromising the defense capacity of the animals against pathogens.

## Figures and Tables

**Table 1:** Putative immune pathways susceptible to be modulated by probiotics but not demonstrated in poultry.

Metabolic Pathway	Known Functions	Putative Role on Inflammation
**Acute Phase Proteins**		
Haptoglobin	↓Free hemoglobin, ↓oxidative damage	Anti-inflammatory
Ceruloplasmin	↓Neutrophils, ↑peroxide scavenging	Anti-inflammatory
C-Reactive protein	Activation of complement	Pro-inflammatory
Mannose-binding lectin	Activation of complement, reduction of CpG-ODN motifs, increase of phagocytosis against pathogens	Pro-inflammatory
**Complement**		
Complement proteins in the epithelium	Induction of phagocytosis, cytolysis, and inflammatory response	Pro-inflammatory
*TLR*		
TLR2 and 4	Detection of LPS, inflammatory response through the NF-κB pathway	Pro-inflammatory
TLR21 basolateral	Detection of CpG-ODN motifs, activation of NF-κB pathway	Pro-inflammatory
TLR21 apical	Detection of CpG-ODN motifs, inactivation of NF-κB pathway	Anti-inflammatory
**Immune cells**		
NK cells	Kill infected cells, cytotoxicity mediation, maintenance of homeostasis, cell barrier integrity	Pro- and anti-inflammatory
γδ T cells	Cytotoxicity, ↑IL17a	Pro-inflammatory
Treg cells	↑Tolerance, ↑IL10, ↑TGF-β, ↑sIgA, ↓hypersensitivity to food	Anti-inflammatory
**Antimicrobial peptides**		
Expression of HDP	↑Antimicrobial activity	

↑ = Increase, ↓ = Reduction.
